# Damage-free plasma etching of porous organo-silicate low-k using micro-capillary condensation above −50 °C

**DOI:** 10.1038/s41598-018-20099-5

**Published:** 2018-01-30

**Authors:** R. Chanson, L. Zhang, S. Naumov, Yu. A. Mankelevich, T. Tillocher, P. Lefaucheux, R. Dussart, S. De Gendt, J.-F. de Marneffe

**Affiliations:** 1 IMEC v.z.w., 75 Kapeldreef, B-3001 Leuven, Belgium; 2 Leibniz-Institut fur Oberflachenmodifizierun, 15 Permoserstrasse, 04318 Leipzig, Germany; 3 Skobeltsyn Institute of Nuclear Physics, Moscow State University, SINP MSU, Moscow, 119991 Russia; 4 GREMI, University of Orleans and CNRS, 45067 Orleans, France; 5 KU Leuven, Celestijnenlaan 200F, B-3001 Leuven, Belgium

## Abstract

The micro-capillary condensation of a new high boiling point organic reagent (HBPO), is studied in a periodic mesoporous oxide (PMO) with ∼34 % porosity and k-value ∼2.3. At a partial pressure of 3 mT, the onset of micro-capillary condensation occurs around +20 °C and the low-k matrix is filled at −20 °C. The condensed phase shows high stability from −50 < T ≤−35 °C, and persists in the pores when the low-k is exposed to a SF_6_-based plasma discharge. The etching properties of a SF_6_-based 150W-biased plasma discharge, using as additive this new HBPO gas, shows that negligible damage can be achieved at −50 °C, with acceptable etch rates. The evolution of the damage depth as a function of time was studied without bias and indicates that Si-CH_3_ loss occurs principally through Si-C dissociation by VUV photons.

## Introduction

CMOS dimensional scaling causes RC delay to increase, leading to slow interconnects for highly scaled logic. Besides the introduction of low-resistivity metals replacing Cu, ultra low-k porous materials (ULK) are considered for the replacement of SiO_2_ (k = 4.2) and dense organo- or fluorinated silicates (k ≥ 3.0). Starting from an organo-silicate matrix, k-values below 2.5 can only be obtained by creating artificial porosity above the percolation threshold (∼20%), leading to largely interconnected pores^1^ Many attempts have been performed for the integration of ULK materials. The usual steps necessary to build interconnects have been extensively studied, from hard mask opening and low-k plasma etching process, to post etch clean, low-k sealing, barrier deposition, copper fill and chemical-mechanical polishing^2–9^, however there is not yet a consensus on the optimal approach for low-damage ULK plasma patterning resulting in significant reduction of the RC delay.

Plasma-induced damage (PID) has been largely studied and has three main sources: chemically active radicals, high-energetic bombardment by charged species and UV-VUV photons^10,11^. Plasma radicals such as O^*^, N^*^, H^*^, ${{\rm{CF}}}_{x}^{\ast }$ can chemically react with methyl bonds, forming hydrophilic silanols which, upon ambient exposure capture moisture and lead to significant k-value increase. The precise radical damage mechanism is rather complex and is influenced by multiple intrinsic (material) and extrinsic (plasma) factors including porosity, pore size and tortuosity, Si/C ratio, methyl density on pore walls (intrinsic), ion/neutral flux, and the presence of CF_*x*_ passivation layer (extrinsic). Ion bombardment has opposite effects, i.e. can cause densification leading to the formation of a top SiO_2_ layer, but also sealing against the propagation of radicals^5^. The effect of plasma-emitted UV-VUV light has been recently the subject of intense investigations, showing that photons with wavelength 100 ≤ *λ* ≤ 200 nm can cause Si-C scissioning deep into the bulk of the materials, but this effect can be significantly reduced by the use of a proper hard-mask system acting against VUV penetration^12^.

Despite these studies, which led to a better understanding of plasma-ULK interactions, at present no simple way has been found to totally avoid plasma damage of the low-k during plasma etching. Many low damage options have been explored up till now^3,13–17^. The first type of approach consists of repairing the organo-silicate material after the etching process, through *in-situ* CH_4_ plasma^18^ or subsequent sylilation^13^ Some restoration may be obtained, but it is partial and is typically restricted to a few nanometers, leading to residual bulk damage. Another method consists of removing the porogen after low-k integration^19^ (post-integration porogen burn-out). This was discontinued due to mechanical failure (ULK-metal delamination caused by film shrinkage during porogen removal). A variation of this approach has been proposed recently^20^ which resolves the mechanical failure by carefully controlling post-deposition bake temperature and time. Frot *et al*.^14,15^ proposed to fill the porous low-k matrix by sacrificial polymers, after complete material synthesis and before further material processing. The polymer is removed after etching or after the metallization is completed (after chemical mechanical polishing). This method has been shown to prevent part of the processing damage, nevertheless it requires multiple additional processing steps and some issues remain to be clarified, from the material perspective and/or for the polymer removal step.

Recently some insight has been made^16,17,21^ on the use of low substrate temperature to reduce damage during plasma etching. The original papers investigated pure SF_6_ and SF_6_/SiF_4_/O_2_ mixtures, then later used C_4_F_8_/SF_6_ for low-k cryogenic etching at around −120 °C. The method consists in condensing, in the pores, reacted by-products or a gas at low temperature in order to protect the material during the plasma etch process. The condensed phase is later desorbed during warm-up to room temperature and/or by additional annealing. This technique shows good results^21^ in terms of damage mitigation as well as electrical yield and pattern transfer (no wiggling nor delamination of hard mask), indicating negligible mechanical damage due to extreme cryogenic cooling (−120 °C). The extreme low temperature needed to condense C_4_F_8_ and protect the Si-CH_3_ bonds remains nevertheless a technical challenge with respect to tool reliability. The protection mechanism, based on pore filling, is driven by micro-capillary condensation of an external reagent, as described in^17^. It results from preferential condensation into nanometer-scale confined geometries, and is usually described by the Kelvin equation or modifications thereof:

1 $$ln\frac{{p}_{g}}{{p}_{sat}}=-\frac{2{\gamma }_{l,g}{V}_{m}}{rRT}$$

where *p*_*g*_ is the vapor pressure of the reagent in confined geometry, *p*_*sat*_(*T*) its saturated vapor pressure, *γ*_*l*,*g*_ is the surface energy between the gas and the liquid, *V*_*m*_ is the molar volume of the condensed liquid, *R* is the universal gas constant, *r* is the radius of the droplet at the liquid-vapor phase boundary, and *T* is the temperature. The capillary condensation is therefore driven by the system temperature, and the reagent vapor pressure, molecular size and surface energy.

Process-friendly conditions are a combination of (1) temperature T ≥ −50 °C, enabling cost-effective hardware design; and (2) precursor partial pressure *p*(*T*) in the mTorr regime at full condensation. The latter will enable low-pressure plasma processing while having small to negligible impact on the plasma chemistry when the discharge is on. The challenge is to find a reagent with saturated vapor pressure *p*_*sat*_(*T*) enabling eq. 1 to be satisfied, above T ≥ −50 °C, while its partial pressure *p* is of the order of a few miliTorr and for pore diameters up to *d*_*max*_ ≈3 nm.

## Methods

The present paper describes the micro-capillary condensation properties and plasma damage mitigation properties of a specific high boiling point organic (HBPO) molecule (provided under tradename *nerima* by Air Liquide, subject to non-disclosure agreement). It is a heavy fluorocarbon; it has its boiling point above 100 °C at atmospheric pressure and shows good wetting of the low-k surface (contact angle <5°). The saturated vapor pressure V_*p*_(*T*) can be estimated using Antoine equation’s in the temperature range [200–340] K, leading to estimated values ranging from V _*p*_(223.15 K) ∼ 12 mTorr to V _*p*_(273.15 K) ∼ 1240 mTorr. The periodic mesoporous oxide films, SBA-2.2 from *SBA materials*, were deposited on 300 mm n-type Si wafers by spin-coating of a solution containing a mixture of organosilica esters and amphiphilic molecules as a template. The molecules consist of linear hydrophilic and hydrophobic blocks represented by polyethylene oxide and a saturated hydrocarbon chain, respectively. After coating and short low temperature annealing in air (soft-bake), the films were sintered at 400 °C in N_2_ for 120 minutes, allowing for the production of a mechanically stable and template free low-k film of approximately 200–210 nm thickness, refractive index of 1.27 (at 633 nm) and measured k-value of 2.31. The plasma etch system used in this work is an Alcatel A601E ICP chamber equipped with liquid N_2_-cooled 6 inch wafer holder, which has been thoroughly described in previous publications^16,17,21^. The plasma etch system is equipped with an *in-situ* spectroscopic ellipsometer from Jobin-Yvon and a liquid injection system composed of a Bronkhorst controlled evaporator mixer connected by flow controllers to the vessel containing liquid HBPO *nerima* and a carrier gas which can be either SF_6_, Ar, C_4_F_8_ or CF_4_. Due to technical constraints, the time-dependent ellipsometry measurements were limited to a maximum rate of one spectrum per 30 s. For condensation and etching studies, coupons of 4 × 4 cm^2^ were glued on a SiO_2_-coated silicon carrier wafer using polyethylene glycol wax. Standard plasma processing is done in two steps using the following conditions: first a condensation step at −50 °C with gas flows Q(SF_6_/HBPO) = 30/4 sccm at 22.5 mTorr (no plasma). Afterwards, in a second step the plasma is ignited with the following parameters P _*rf*_ = 500 W, P_*DC*_ = 150 or 0 W bias, p = 22.5 mTorr. In this work, substrate temperature, etch time and applied bias were the main variables. After plasma processing, the low-k films were annealed at 400 °C for 10 min under N_2_ atmosphere. No damage was observed in the pristine material after this annealing. Films were characterized by spectroscopic ellipsometry (Sentech SE801) for refractive indices and thickness (single layer Cauchy model). The chemical changes were determined by means of transmission FTIR spectroscopy (Nicolet 6800); the damage is determined by the change of relative intensities of the Si-CH_3_ (methyl bonds) and Si-O-Si peak (matrix). This variation is mathematically described by the so-called *equivalent damage layer* (EDL) described in eq. 2 with *ρ* as the Si-CH_3_/Si-O-Si peak ratio and *d* the thickness of the low-k film^22^. The depth profile of the damage was evaluated by TOF-SIMS depth profiling (TOFSIMS IV from ION-TOF GmbH operating in dual beam configuration). Dielectric constant of the low-k films was determined from capacitance measured with an E4980A precision LCR meter on planar metal-insulator-semiconductor structures formed by the evaporation of 70 nm Pt contact pads on top of n-Si/low-k stacks.

2 $$EDL=d(1-\frac{{\rho }_{measured}}{{\rho }_{pristine}})$$

## Results

### Reagent condensation and stability of the condensed phase

**Figure 1:**
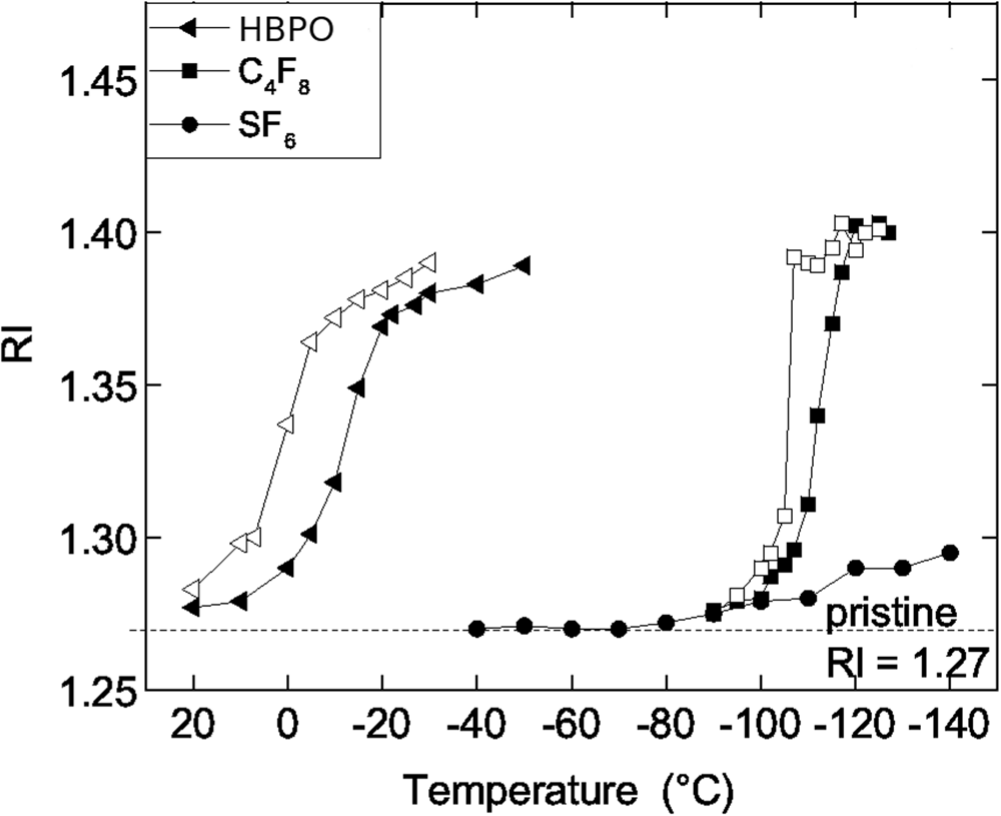
Adsorption-desorption cycles in temperature with Q(SF_6_/HBPO) = 30/4 sccm at p = 22.5 mTorr and with reference gases C_4_F_8_ and SF_6_ at p = 7.5 mTorr. Adsorption is represented by full symbols and desorption by open symbols.

Figure 1 shows the *in-situ* change of the low-k’s refractive index (RI), measured as a function of temperature when exposed to a gas flow (Q(SF_6_/HBPO) = 30/4 sccm, Q(SF_6_) = 30 sccm or Q(C_4_F_8_) = 30 sccm) at partial pressure of reagent at *p* = 2.5 mTorr (for HBPO) and *p* = 7.5 mTorr for SF_6_ and C_4_F_8_ alone. At room temperature, the measured refractive index (RI) of 1.27 corresponds to the pristine low-k film, with porosity 34%. By decreasing the temperature, the RI increases indicating the onset of micro-capillary gas condensation. Using the Lorentz-Lorenz equation and the HBPO’s RI (1.275 around 20 °C), it can be calculated that for a measured RI = 1.378, the pores become ∼99.5% filled. For C_4_F_8_, with a RI of 1.45, the total filling occurs around −120 °C with a RI of 1.41. A hysteresis cycle is observed, which is typically attributed to the inversion of the liquid meniscus between absorption and desorption into the micropores, leading during desorption to a local effective pressure higher than the global pressure in the reactor (the so-called Laplace pressure).

**Figure 2:**
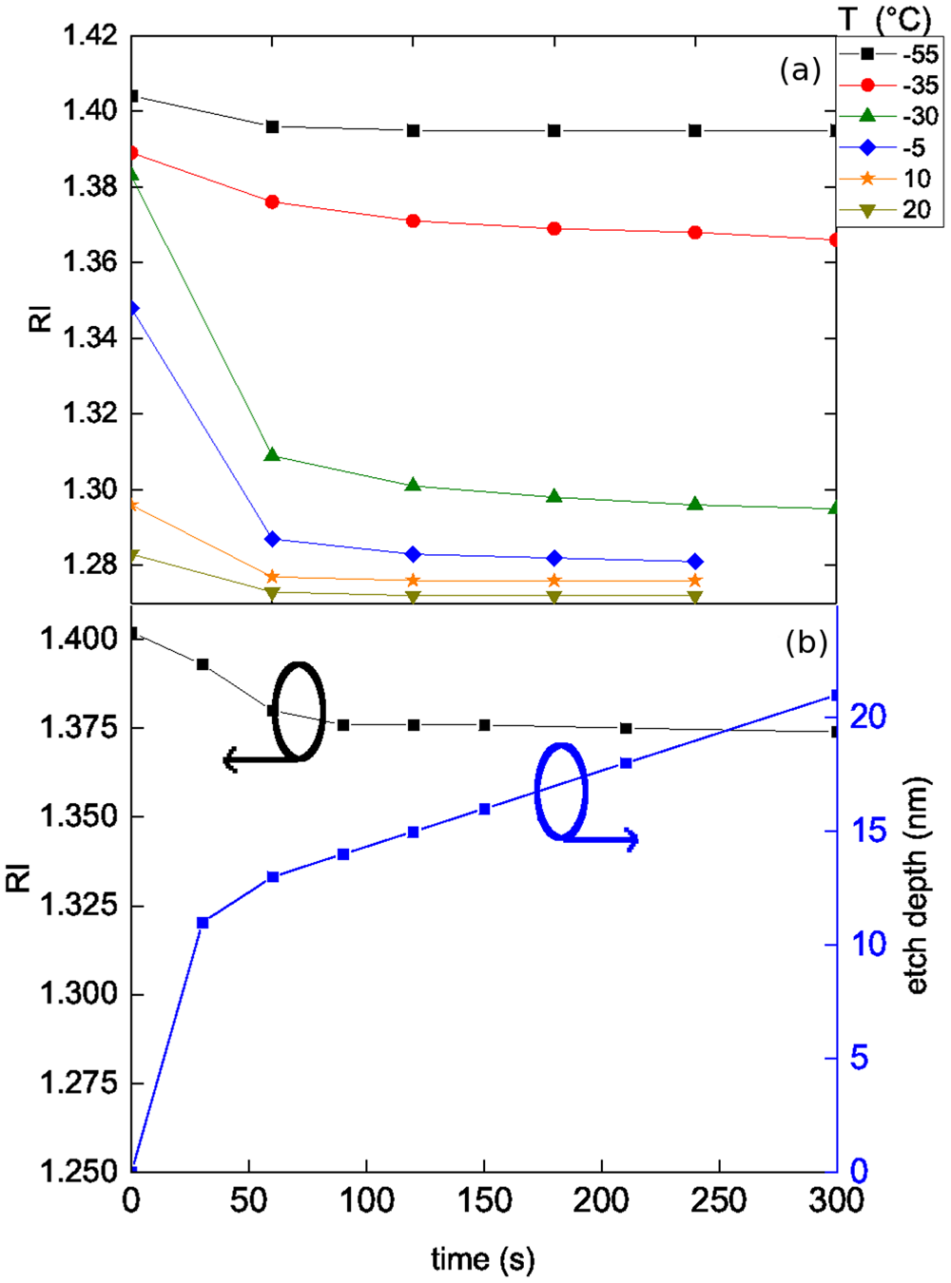
(**a**) Condensate stability with temperature. At each temperature, the HBPO is condensed (RI measured at 0 min) with Q(SF_6_/HBPO) = 30/4 sccm and p = 22.5 mTorr; then, the HBPO is pumped out and the RI is measured every min for 5 minutes; (**b**) Condensate stability with pure SF_6_ plasma. The HBPO is condensed at T = −50 °C, p = 22.5 mTorr, Q(SF_6_/HBPO) = 30/4 sccm. Then the HBPO is pumped out and plasma is turned on with P_*r*_*f* = 500 W and P_*DC*_ = 0 W bias.

In order to better characterize the condensed phase stability, tests were performed according to the following sequence. At different fixed temperatures, the reagent is condensed for 1 minute; then, the reagent flow is stopped (only SF_6_ carrier gas remains flowing in the reactor) and the RI is measured as a function of time. The resulting isotherms are shown in Fig. 2(a). From 20 to 10 °C the RI drops back to 1.27 indicating that the condensed product is fully desorbed. For lower temperatures, between −5 and −30 °C, after pumping the reagent, the RI drops to 1.29 and 1.31 respectively and remains stable after 5 minutes. This indicates an incomplete restoration of the porosity, i.e. despite bulk HBPO desorption some layer(s) of condensate remain adsorbed at pore walls. Between −35 to −55 °C, the RI remain high after pumping out the HBPO. The slight decrease of RI during the first minute is due to the desorption of the gas-liquid interfacial layer. The slight increase of RI from −35 °C to −50 °C is due to higher densification of the condensed reagent when temperature decreases (see also Fig. 1). Below −55 °C, fitting by a Cauchy model was impossible, even by adding a carbon layer over the modeled stack. The layer formed becomes optically non homogeneous, and corresponds to the formation of a condensed liquid film on top of the low-k film. At ∼3 mTorr partial pressure, the system is getting close to or crossing HBPO’s *V*_*p*_(*T*) curve. In the rest of the paper, this state will be called the “over-condensation” state. Our experiments show that the presence of liquid HBPO film on top of the low-k film provides a full protection/encapsulation of the low-k during plasma exposure, which can be fully desorbed at warm-up (no plasma-induced HBPO cross-linking or densification); therefore over-condensation must be avoided so as to enable the etching process.

It can be concluded that there is a stability window between −35 to −55 °C (before entering the “over-condensed” mode) in which the condensed phase remains for a long time in a glassy state; this corresponds to a liquid-solid transition which occurs slightly below this temperature range for the HBPO used in this work (private communication from Air Liquide). Below −30 °C, condensation is therefore replaced by deposition of solid HBPO directly from the gas phase. This is likely possible if we consider the confinement of condensate on the structure^23–29^. The desorption mechanism is then driven, below −30 °C, by sublimation. For the present study, the phenomenon is amplified since in the porous matrix the chains are more confined than on a surface: The liquid-solid transition may occur at higher temperature in confined space^26^.

The stability of the condensed HBPO phase, during plasma processing, has been checked by exposure to an SF_6_-based discharge. For this, HBPO is condensed using the standard conditions at −50 °C. Before the plasma ignition, the HBPO is removed from the gas phase, by turning the mass flow controller off, then the sample is exposed to a pure SF_6_ plasma (P_*rf*_ = 500 W, P_*DC*_ = 0 W bias, p = 22.5 mTorr), still at −50 °C, and the RI is measured every 30 s. As observed in Fig. 2(b), within the first 60 seconds the RI decreases from 1.402 down to 1.375 then stays stable around this value for the next 4 minutes. This phenomenon is not very different from what is observed without plasma, since the plateau in RI corresponds to what is observed around −40 °C in Fig. 2(a). A slight increase of the temperature of a substrate exposed to a plasma is well known, and is expected to lead to further desorption of the nanometer-range over-condensed HBPO film present on top of the PMO below −50 °C. The first 30 s of exposure shows also 10 nm of enhanced low-k removal after which a steady-state is reached with constant etch rate. This indicates the existence of a transient state during which the HBPO protection of the top of the low-k film is likely weakened, which might be caused by the sudden heat and photon flux coming from the plasma. After etch, by pumping out all the gas and warming up to room temperature, the refractive index decreases to 1.337 and not to the pristine value (1.27). After annealing, a RI of 1.28 is recovered. A first hypothesis is a denser low-k matrix, as observed in case of ion-induced damage or porogen removal, due to higher density of Si-O-Si bonds; however it is ruled out since the low-k is restored by annealing. Another option is that the ULK matrix keeps its original RI but some by-products remain inside the low-k film after processing, i.e. the plasma exposure lead to by-products inclusion. These are then removed by annealing, restoring the original RI.

An additional experiment (not shown here) was performed in order to determine the detrimental effect of condensation/decondensation of the HBPO in the porous dielectric material, by exposing the low-k film to 10 cycles at −20 °C (one minute at 100% filling). FTIR spectra indicate that no modification of the low-k material occurs during these cycles. So, the condensation process does not cause irreversible chemical modification of the ULK.

### Processing using biased SF_6_/HBPO plasma

**Figure 3:**
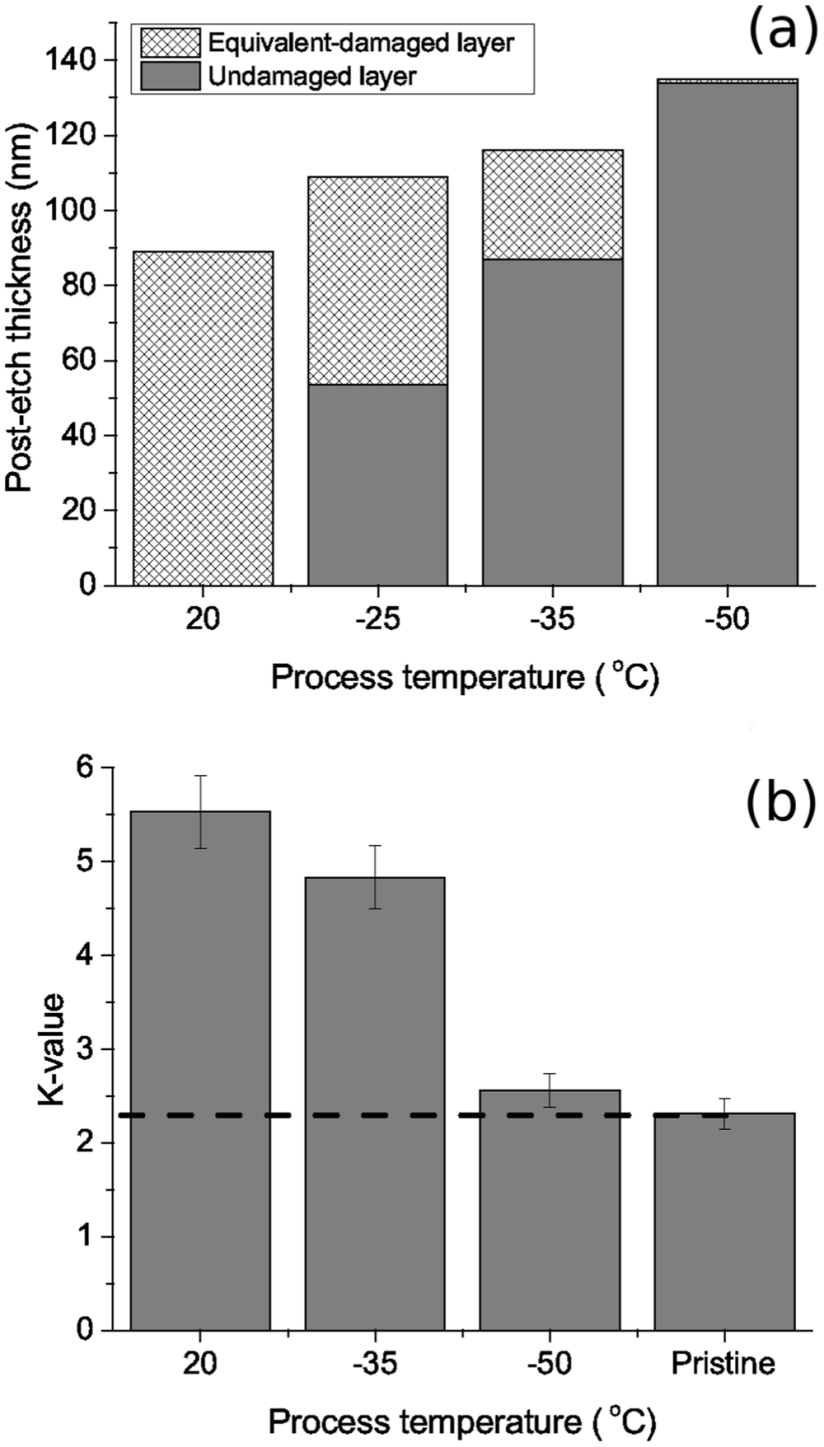
(**a**) Evolution of film thickness and equivalent damage layer after 60 s etch using p = 22.5 mTorr, Q(SF_6_/HBPO) = 30/4 sccm, P_*rf*_ = 500 W and P_*DC*_ = 150 W bias then annealing for 10 min at 400 °C in N_2_ (the original thickness was 212 nm); (**b**) Extracted k-value for the different conditions; the dashed line corresponds to the pristine k-value.

Figure 3(a) shows the change in equivalent damage layer (EDL) for coupons etched at various temperatures between 20 °C and −50 °C with a bias power of 150 W for 1 minute. At room temperature, the sample is fully damaged, i.e. the Si-CH_3_ bonds are fully removed from the sample and likely the porous structure has partly collapsed. Table 1 shows the RI values after etching and annealing, the RI at 20 °C is 1.305, at −35 °C it drops to 1.298 and down to 1.279 at −50 °C; indicating that the condensate helps protecting from matrix densification by ion bombardment, which typically occurs after huge methyl depletion. By decreasing the temperature, the EDL is reducing and becomes negligible at −50 °C. This is well correlated to the Fig. 3 (b) showing the k-value evolution after plasma exposure in comparison to pristine (k value is measured after annealing at 400 °C). This is correlated to a drop of etch rate (see Table 1), indicating that the etched film is denser, i.e. there is a condensed phase in addition to the low-k matrix (as also observed in the case of C_4_F_8_ condensation^17^). The k-value of these samples has been extracted and shows major improvement for the sample processed at −50 °C.

**Table 1:** Low-k film evolution with temperature after plasma exposure and annealing.

Temperature	RI after annealing	Etch rate (nm.min^−1^)
Pristine	1.27	—
20	1.305	123
−25	Not measured	103
−35	1.298	96
−50	1.279	77

**Figure 4:**
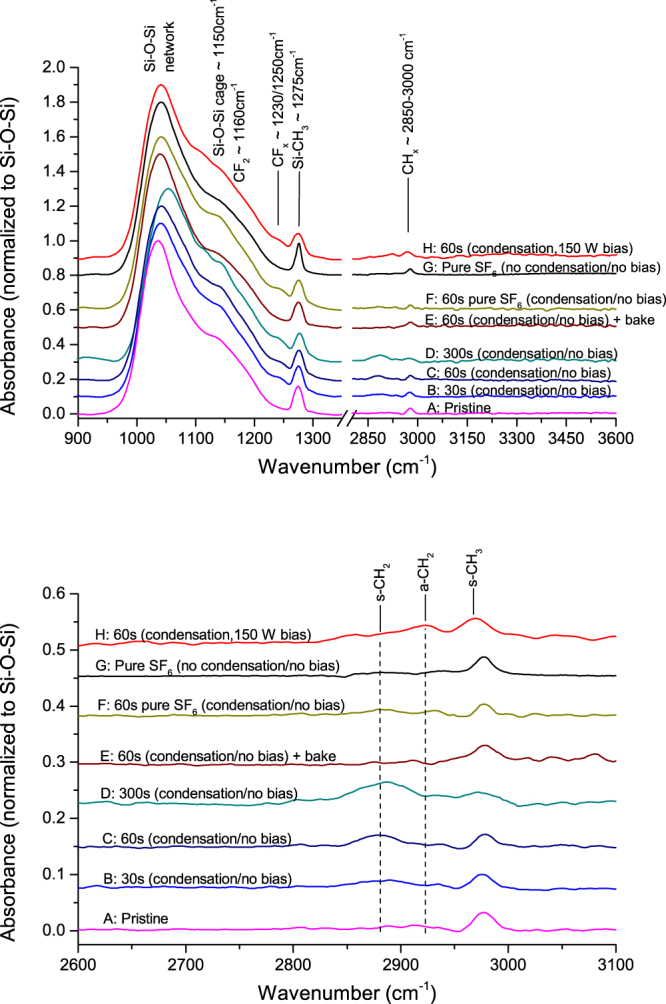
FTIR absorbance spectra for various etch conditions using baseline: Q(SF_6_/HBPO) = 30/4 sccm, t = 60 s, P_*rf*_ = 500 W, P_*DC*_ = 0 W bias, T = −50 °C, no annealing. For selected samples, prior to plasma ignition, the HBPO molecule was condensed into the porous low-k for 60 seconds so as to suppress damage by fluorine radicals. Spectra are normalized to the Si-O-Si peak at ∼1050 cm^−1^.

**Figure 5:**
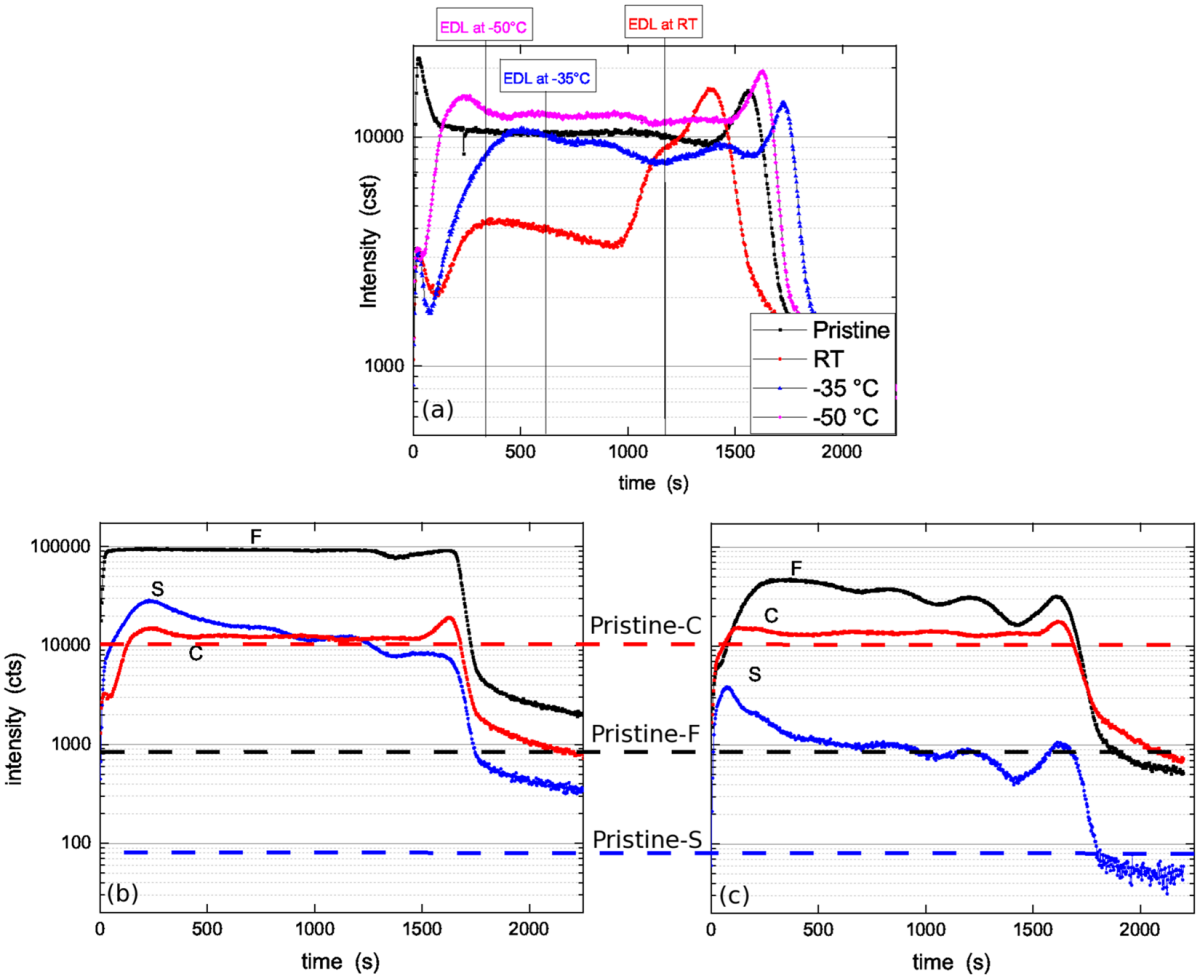
(**a**) Evolution of the Carbon profile after 5 min exposure of a 200 nm low-k film to a SF_6_/HBPO plasma (without bias), at three processing temperatures, without annealing. The EDL position is estimated following the measured residual thickness and EDL of the film after exposure and considering a constant sputtering yield during all the Tof-SIMS analysis; (**b**) C, S and F profiles of the low-k film after plasma exposure without bias F signal was saturated, *before* annealing; (**c**) C, S and F profiles of the low-k film after plasma exposure without bias, *after* annealing.

Figure 4 shows the FTIR spectra of low-k films exposed to various low temperature plasma treatments using *in-situ* HBPO condensation. After SF_6_/HBPO etch without applied bias (spectra B, C and D), there is negligible moisture absorption and the Si-O-Si band shows the appearance of two sub-peaks around 1160 cm^−1^ and 1230–1250 cm^−1^, which can be attributed to CF_*x*_ compounds. These peaks do originate from residues of the HBPO molecule, which contains C and F. The low moisture absorption is in agreement with Fig. 5(a) showing that the carbon profile is well preserved after plasma exposure when temperature decreases. Figure 4 shows also that the Si-O-Si network peak presents a slight shift towards higher wavenumber, indicating some matrix deformation. The Si-CH_3_ is preserved, and the absence of Si-H stretch at 2260 cm^−1^ indicates the absence of H* (coming from the dissociation of methyls) or that dangling Si- bonds can be saturated must faster by other elements (F). Looking at the CH_*x*_ band in the 2800–3000 cm^−1^ range, the CH_3_ peak (2975 cm^−1^) decreases at the benefit of the s-CH_2_ peak (2875 cm^−1^), which is normally attributed to *sp*^3^ CH_2_ (∼2875 cm^−1^)^30^, indicating some H abstraction; however we believe it is caused by some resonance shift due to fluorine. Due to fluorination of the porous low-k, Mankelevich *et al*.^31^ proposed the formation of a Si pentavalent state leading to stretching as observed in the FTIR spectra. This is a first step of a three-stage process involving first fluorination through the insertion of fluorine adatom and formation of OSi(F)CH_3_, second hydrogen abstraction (stepwise transformation of -CH_3_ into -CH_3−*x*_F_*x*_ and volatile HF) and third etching by reaction with the Si-O-Si skeleton. The observation of a *sp*^3^ CH_2_ (∼2875 cm^−1^) after plasma exposure indicates that, for this very long condition, fluorination leading to hydrogen abstraction is initiated in the bulk low-k. This phenomenon is likely to happen in these samples, since, as shown by TOF-SIMS data (Fig. 5(b)) our samples are heavily loaded by F after etch. The FTIR spectrum E shows that after annealing, which lead to fluorine desorption (see Fig. 5(c)), the CF_*x*_ are removed and the CH_3_ peak at 2975 cm^−1^ is restored, indicating that the additional peak at ∼2875 cm^−1^ is likely caused by the formation of OSi(F)CH_3_. The curve F shows the FTIR spectrum obtained after condensation then etched with a pure SF_6_ plasma; it shows still negligible moisture absorption but the additional CF_*x*_ peaks remains present indicating the presence of HBPO residues after etch. The curve G is a reference process etched at −50 °C with pure SF_6_ and no HBPO condensation: some slight moisture adsorption can be observed, indicating that part of the damaged Si-CH_3_ have formed silanols. The curve H shows the FTIR spectrum after etch with 150 W bias (no annealing), indicating again the presence of HBPO residues causing network stretching, and some CH_*x*_ reorganization.

### Processing using unbiased plasma conditions

To etch patterns (90 nm deep trenches) at 150 W bias, typically an etch time of 60 s would be considered, including 30% over-etch. In order to get some insight into the damage mechanism, some further analysis has been made on samples etched at −50 °C in plasma conditions described in Fig. 3. In order to simulate the situation observed at a feature sidewall, which is not exposed to particle bombardment but mainly to isotropic radicals, no bias was applied. We have to keep in mind that the damage which will be observed in this experiment will be more significant in comparison to the sidewall of a patterned structure for two reasons. First, the solid angle is higher for a blanket surface than for the sidewall of a patterned structure; second, there is no partial protection by resist or a hard-mask. In order to amplify the phenomenon, the etch time was extended up to 600 s, way beyond what would be necessary for pattern transfer of a real structure.

**Figure 6:**
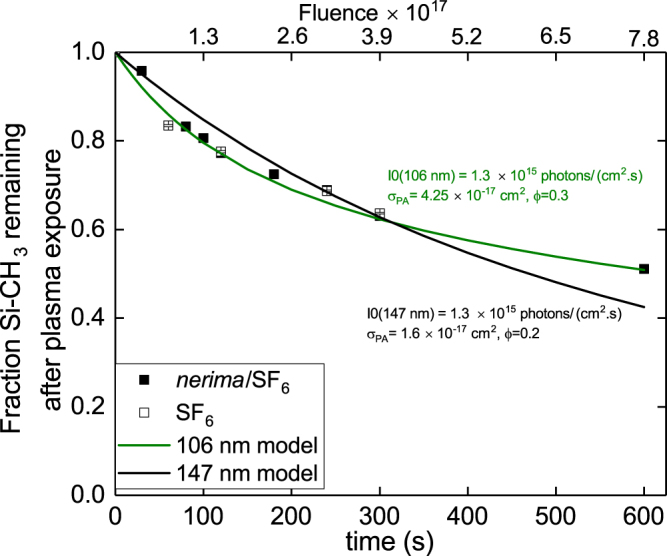
Fraction of Si-CH_3_ depletion in comparison to pristine, etch depth is considered as part of material for which the Si-CH_3_ were removed. Full symbol represent the film exposed to HBPO/SF_6_ plasma and the open symbol represent the film exposed to pure SF_6_ plasma after HBPO condensation. The curves represent the calculated damage induced by VUV emission for two photons fluxes *I*_0_ = 1.3 × 10^15^ at 106 and *I*_0_ = 1.3 × 10^15^ photons/(cm^2^⋅s) at 147 nm (for the VUV model details see section III).

Figure 6 shows the time dependence of the Si-CH_3_ depletion (equivalent to the EDL), for pure SF_6_ and SF_6_/HBPO discharges applied after HBPO condensation. The EDL evolution is the same with or without HBPO in the gas phase. Because the EDL evolution is similar, we can conclude that, once the low-k is filled by the condensed phase, the presence of HBPO in the plasma (gas) phase affects only marginally the level of damage in the low-k, i.e. the condensed phase does not vaporize and is not etched away by the SF_6_ plasma species. Moreover, damage propagates in the same way for a pure SF_6_ plasma and a SF_6_/HBPO plasma mixture.

Figure 5(a) shows the change in Carbon profile after 5 minutes exposure to the non-biased SF_6_/HBPO plasma, for three different processing temperatures. At room temperature, there is strong C depletion over more than half the sample thickness, indicating strong damage. By decreasing the temperature down to −50 °C, the damaged part reduces significantly. However, at −50 °C the carbon content in the bulk increases slightly, which correlates with CF_*x*_ residues as found by FTIR. Figure 5(b) and (c) show the effect of annealing on the C, F and S profiles, compared to pristine, for the sample processed at −50 °C. First, it can be seen that even by using HBPO condensation, the level of fluorine into the low-k raises by minimum two orders of magnitude (actually the F signal was saturated in Fig. 5(b)); by annealing this can be slightly decreased but the pristine level cannot be restored. A similar observation is made for sulfur. As explained previously, the presence of F can be explained either by HBPO photodissociation or diffusion of plasma radicals, through the condensed phase; it is clear that the high S level in the samples indicates that in-diffusion from plasma species is non-negligible. Annealing allows to decrease the global level of F and S, in the bulk and at surface level. Within experimental accuracy, annealing does not cause major change in the carbon profile, indicating some residual carbon in the film.

### Photo-dissociation of the HBPO molecule

**Figure 7:**
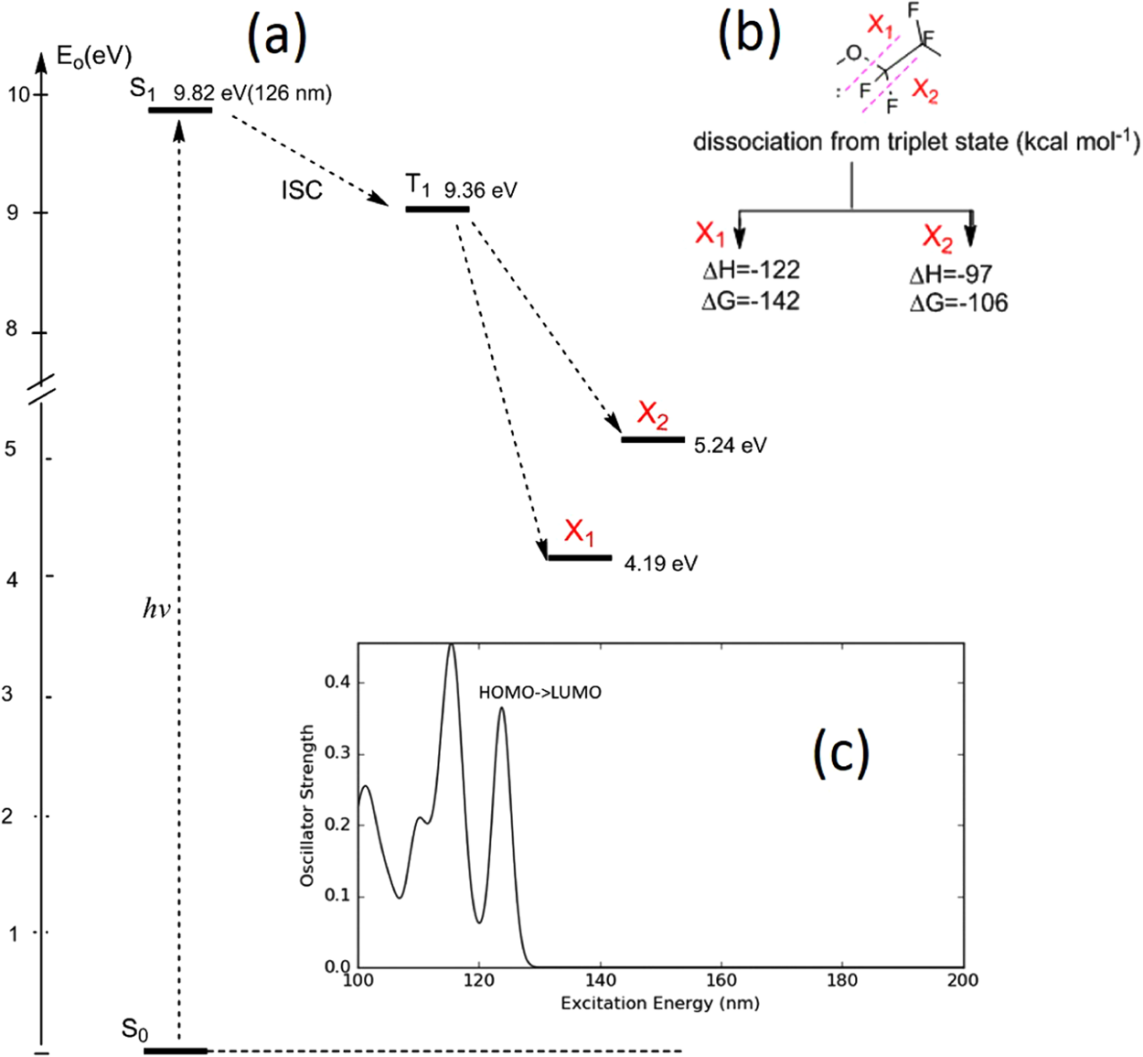
Results of DFT quantum chemical calculations: (**a**) energy scheme of the radicals formation after excitation; (**b**) most energetically favorable bonds dissociation reactions X_1_ and X_2_ as calculated for the HBPO molecule. Here ΔH and ΔG (given in kcal/mol) are calculated starting from the optimized triplet T1; (**c**) calculated absorption VUV spectra of HBPO.

Quantum chemical calculations were performed using the density functional theory (DFT) B3LYP method^32,33^ (Jaguar, version 9.6)^34^ to study VUV spectra and energetics of reactivity of the HBPO molecule. The structure of the molecule was optimized at B3LYP/6-31(d,p) level. The frequency analysis was made at the same level to obtain thermodynamic parameters such as total enthalpy (H) and Gibbs free energy (G) at temperature according to experimental conditions, namely −50 °C. The reaction enthalpies (ΔH) and Gibbs free energies of reaction (ΔG) were calculated as the difference of the calculated total enthalpies and Gibbs free energies between the reactants and products respectively. The electronic transition spectra were calculated using the time dependent^35^ (TD) DFT method at B3LYP/6-31 + G(d,p) level. The results of the calculation are shown in Fig. 7 (due to confidentiality reasons, the entire molecule cannot be shown). The energy scheme shows the formation of a triplet state T_1_ after absorption of a photon of 9.82 eV (*λ* = 126 nm), which dissociates according to reactions X_1_ and X_2_ (see Fig. 7(b) for more details). Reaction X_2_ keeps the original molecule size but results in the abstraction of one fluorine atom. Reaction X_1_ leads to molecule scission into two sub-molecular radicals of equivalent size, which can be further photo-dissociated into smaller fragments. Reaction X_1_ is energetically more favorable than X_2_, however we cannot exclude the release of F^*^ radicals into the porous low-k, that can react with Si-CH_3_ bonds or reaction products from X_1_. Nevertheless, the detrimental effect of these by-products has been shown to be reversible after annealing at 400 °C and/or negligible in regard to VUV damages.

## Discussion

In the previous section, the micro-capillary condensation of a HBPO fluorocarbon molecule has been demonstrated, leading to complete pore filling below −30 °C at a partial pressure of ≈3 mT. The condensed phase, in the absence of gas phase (HBPO evacuated from the chamber after condensation), has been shown to be stable for several minutes, for temperatures below −35 °C. From −55 °C, some over-condensation occurs which is passivating all surfaces. In the presence of plasma, the condensed phase remains stable in the bulk; only the surface shows indication of HBPO depletion during the first seconds of plasma onset.

After HBPO condensation, by exposing the low-k samples to a SF_6_/HBPO 150 W-biased plasma, it is shown that, around −50 °C, the Si-CH_3_ depletion is below experimental accuracy as well as measured k-values drift, while keeping a reasonable etch rate (77 nm/min). FTIR chemical analysis shows the presence of CF_*x*_ and CF_2_ residues after plasma processing, which most can be removed by 10 minute annealing at 400 °C under N_2_. These residues originate from the HBPO molecule itself, likely degraded during plasma processing.

By exposing the low-k film for long time under a non-biased SF _6_/HBPO plasma, damage still occurs, as shown in Fig. 6. A rough estimate indicates that the damage rate (change in EDL as a function of time) is ≈15 nm/min, to be compared to an etch rate of 77 nm/min (150 W bias). This shows that the etch rate is significantly faster than the damage rate, which enables low damage processing. The damage profile, as shown in Fig. 5, shows C depletion curves which demonstrate the protecting impact of HBPO condensation, and confirm the presence of CF_*x*_ residues which can be partly removed by annealing.

Besides sputtering-induced low-k densification which is assumed negligible in the present case, the two other causes of bulk low-k damage are chemical reactions between the plasma reactive radicals (F^*^) and/or its VUV emission. It was shown in Fig. 5(b) that fluorine penetrates very deeply in the low-k film. On one hand, as shown in previous publications^36^, fluorine penetration cannot be fully suppressed by pore filling due to the high mobility of F into polymers. A second source of fluorine is the plasma-induced dissociation of the condensed HBPO phase, leading to the release of active fluoro(carbon) species in the bulk of the low-k. Despite this high fluorine load deep in the bulk, carbon depletion occurs only on the top. So, fluorine radicals have a negligible effect on the methyl depletion mechanism in our case. As a consequence, VUV emission seems to be the most important contributor to damage. According to Rakhimova *et al*.^37,38^, VUV emission between 100 nm and 190 nm is the most damaging for Si-CH _3_ bonds in the bulk low-k material. As shown in literature^12,39^, pure SF_6_ does emit in the VUV range.

We can therefore propose the following mechanism for ULK modification by exposure to SF_6_/HBPO plasma in the presence of a condensed HBPO phase. First, the amount of fluorine incorporation in the bulk, found in our experiments, is surprisingly large, in view of the fact that the condensed phase remains during plasma processing, as shown by the Fig. 1(c) and the nice preservation of the k-value at −50 °C. In the case of the pore stuffing approach, which consists of using low molecular weight polymer (PMMA, PS) for protecting against plasma damage, the level of fluorine incorporation is much lower and even suppressed deep into the bulk^36^. Another reference is the use of C_4_F_8_ as reagent where, similarly to this work, the level of fluorine diffusion is only marginally lowered by condensation^17^. Organic molecules are sensitive to UV/VUV light and can undergo, under photon flux emitted by the plasma discharge, various kind of chain scissioning or bond breakage: C=O lactone bonds in resists^40^, bulk depolymerization and oxygen depletion reactions in styrene-based and ester-based model polymers^41^, C-C bond scission in PMMA, PS and PEG^36^. The HBPO molecule is a long fluorocarbon chain which is not an exception to this rule, i.e. due to plasma VUV light, it will get dissociated, likely releasing active F⋅ into the structure; in addition it contains bonds which are particularly sensitive to photodissociation, as shown by DFT calculation (see Fig. 7). In open pores, the damage propagation follows a (*t*)^1/2^ dependency, as observed with different reactive plasma in^5,6,8,17^. The EDL evolution is described then by the random walk theory, which assumes that the reactant has a mean free path longer than the pore diameter. In the present case, with a condensed liquid/solid phase filling the pores, this mechanism does not hold. This is confirmed by the fact that the profile of carbon does not show major Si-CH_3_ depletion despite the fact that fluorine (and sulfur) is massively present in the bulk.

To enlighten VUV as main source of damage, the damage propagation shown in the Fig. 6 can be estimated using VUV photo-dissociation calculations. Damage evolution by VUV is modeled using the equation^37^:

3 $$[C{H}_{3}(t,z)]={[C{H}_{3}]}_{0}\cdot \exp (-t\cdot {\sigma }_{PA}\cdot {\varphi }\cdot {I}_{0}\cdot \exp (-{\sigma }_{PA}\cdot [Si]\cdot z))$$

where *σ*_*PA*_ is the photo-absorption cross section, *ϕ* is an adjustment parameter allowing to consider the effective *CH*_3_ out-diffusion, [Si] is the Si concentration into the low-k, and *I*_0_ is the radiation intensity. Typical SF_6_ plasma emission spectrum in VUV range is shown in references^12,39^. The continuous emission in 100 nm to 150 nm range with local maxima (e.g. at 147 nm) is capable to damage Si-CH_3_ groups, as it was detected in reference^42^ for atomic lines at 13.5, 58.4, 106, 147 nm. The maximal photo-absorption cross-sections for various low-k films were obtained for VUV irradiation at 58.4 and 106 nm for He and Ar atomic lines, respectively^37^. Photo-absorption cross-sections decreased at larger wavelengths: ArF laser irradiation at 193 nm barely damaged low-k films^37,42^. Thus we can expect that the highest peaks of SF_6_ plasma intensity in 185–195 nm range will be probably much less detrimental to the low-k material.

If we assume that the experimental Si-CH_3_ damage is determined only by VUV photons, we can derive an estimate for the total VUV intensity I_0_ in the most damaging range. Photo-absorption cross-sections and quantum yields for Si-C bond breaking (*σ*_*PA*_ = 4.25 × 10–17 cm^2^, *ϕ* = 0.3 at 106 nm VUV emission and *σ*_*PA*_ = 1.6 × 10–17 cm^2^, *ϕ* = 0.2 at 147 nm) deduced for identical ULK films (SBA-2.2) and the model of VUV induced damage^42^ allows us to obtain the absolute level and possible variations of intensity I_0_ in the pure SF_6_ and SF_6_/HBPO plasmas. It turns out that intensities I_0_ on the surface of low-k films are localized in the same range as 1.3 × 10^15^ photons/(cm^2^s) for the used (*σ*_*PA*_, *ϕ*) values at 106 and 147 nm (see fitted curves for SF_6_ and SF_6_/HBPO plasmas, Fig. 6). Therefore, experimental and model data indicate the level of total intensities in the range of VUV irradiation capable to destroy Si-CH_3_ surface groups in the studied film. As expected, these intensities are very similar for SF_6_ and SF_6_/HBPO plasmas. The emission at 106 nm with equation 3 is fitting well the methyl depletion contrary to the one centered around 147 nm; this is where SF_6_ plasma does emit, together with multiple impurities/contaminants such as O and N, as typically found in dielectric processing plasma^39^. According to Fig. 7, the global simulated adsorption spectrum of the HBPO shows strong absorption of the VUV between 100 to 130 nm. Since the *σ*_*PA*_ and *ϕ* are the one used for SBA2.2 with open pores (no HBPO condensed phase), the fluence able to fit the Si-CH_3_ depletion underestimate the true emission because the condensed HBPO absorb a part of the VUV emission.

## Conclusion

A new high boiling point fluorocarbon etch gas (HBPO), allowing plasma etching at a relatively high temperature (higher than −50 °C) was investigated. Condensation properties and stability were studied: full micro-capillary condensation is observed from −20 °C at 3 mTorr partial pressure, and a stability window is found between −35 and −55 °C, most likely related to the presence of a glassy/solid HBPO phase in the pores. In this temperature and pressure range, during exposure to a pure SF_6_ plasma, the HBPO remains in a condensed phase for at least 5 minutes. The condensed HBPO does protect the low-k during plasma etching at −50 °C, with relevant etch rate, leading to negligible Si-CH_3_ depletion and post-etch k-value close to pristine value.

Considering −50 °C processing with an SF_6_/HBPO plasma, FTIR and TOF-SIMS show that CF_*x*_ and F by-products are present into the low-k after plasma exposure, which can be removed by annealing. Quantum chemical calculations indicate that these residues originate from the partial photo-dissociation of the HBPO by SF_6_ plasma VUV energetic photons. Nevertheless, methyl depletion remains low in the bulk, i.e. occurs only on the top of the material when the HBPO is present into the low-k. Chemical reactions with F* or CF*_x_ radicals are therefore not the main source of methyl depletion. The most relevant explanation is plasma VUV emission, since SF_6_ plasma emits between 100 to 190 nm, which is the most sensitive range for photo-induced Si-C scission. It is found that the emission around 106 nm is the main cause of methyl depletion. Quantum chemical calculations show that the HBPO absorb between 100 to 130 nm the VUV emission and so partly protect the low-k from VUV damage. The role of sulfur, which appear as post-etch contaminant in the films, could not be elucidated in this study, due to the lack of evidence for S-based chemical bonds observed by FTIR.

We believe that the present results open a new route for low-damage plasma patterning of ultra-low-k porous dielectrics, solving a decade-old issue which led to multiple shifts of the interconnects ITRS roadmap in the last fifteen years.
